# Veno-Arterial Extracorporeal Membrane Oxygenation Rescue in a Patient With Pulmonary Hypertension Presenting for Revision Total Hip Arthroplasty: A Case Report and Narrative Review

**DOI:** 10.7759/cureus.28234

**Published:** 2022-08-21

**Authors:** Ailan Zhang, Virgilio De Gala, Peter W Lementowski, Draginja Cvetkovic, Jeff L Xu, Andrew Villion

**Affiliations:** 1 Department of Anesthesiology, Westchester Medical Center/New York Medical College, Valhalla, USA; 2 Department of Orthopedic Surgery, Westchester Medical Center/New York Medical College, Valhalla, USA

**Keywords:** regional anesthesia, noncardiac surgery, perioperative management, right ventricular failure, extracorporeal membrane oxygenation, pulmonary hypertension

## Abstract

Patients with pulmonary hypertension (PH) are at an increased risk of perioperative morbidity and mortality when undergoing non-cardiac surgery. We present a case of a 57-year-old patient with severe PH, who developed cardiac arrest as the result of right heart failure, undergoing a revision total hip arthroplasty under combined spinal epidural anesthesia. Emergent veno-arterial (VA) extracorporeal membrane oxygenation (ECMO) was undertaken as rescue therapy during the pulmonary hypertensive crisis and a temporizing measure to provide circulatory support in an intensive care unit (ICU). We present a narrative review on perioperative management for patients with PH undergoing non-cardiac surgery. The review goes through the updated hemodynamic definition, clinical classification of PH, perioperative morbidity, and mortality associated with PH in non-cardiac surgery. Pre-operative assessment evaluates the type of surgery, the severity of PH, and comorbidities. General anesthesia (GA) is discussed in detail for patients with PH regarding the benefits of and unsubstantiated arguments against GA in non-cardiac surgery. The literature on risks and benefits of regional anesthesia (RA) in terms of neuraxial, deep plexus, and peripheral nerve block with or without sedation in patients with PH undergoing non-cardiac surgery is reviewed. The choice of anesthesia technique depends on the type of surgery, right ventricle (RV) function, pulmonary artery (PA) pressure, and comorbidities. Given the differences in pathophysiology and mechanical circulatory support (MCS) between the RV and left ventricle (LV), the indications, goals, and contraindications of VA-ECMO as a rescue in cardiopulmonary arrest and pulmonary hypertensive crisis in patients with PH are discussed. Given the significant morbidity and mortality associated with PH, multidisciplinary teams including anesthesiologists, surgeons, cardiologists, pulmonologists, and psychological and social worker support should provide perioperative management.

## Introduction

There is an increased risk of perioperative morbidity and mortality for patients with pulmonary hypertension (PH) undergoing non-cardiac surgery [1-3]. Right heart failure, prolonged mechanical ventilation, increased post-operative pulmonary complications, stroke, myocardial ischemia/infarction, arrhythmia, respiratory failure, sepsis, and renal insufficiency have been reported as major adverse events [4-6]. We report a case of a 57-year-old patient with severe PH who underwent a revision total hip arthroplasty under combined spinal-epidural (CSE) anesthesia. Emergent veno-arterial (VA) extracorporeal membrane oxygenation (ECMO) was placed as a rescue therapy during pulmonary hypertensive crisis and served as a temporary support [7,8]. We present a narrative review on perioperative management for patients with PH undergoing non-cardiac surgery.

## Case presentation

**Figure 1:**
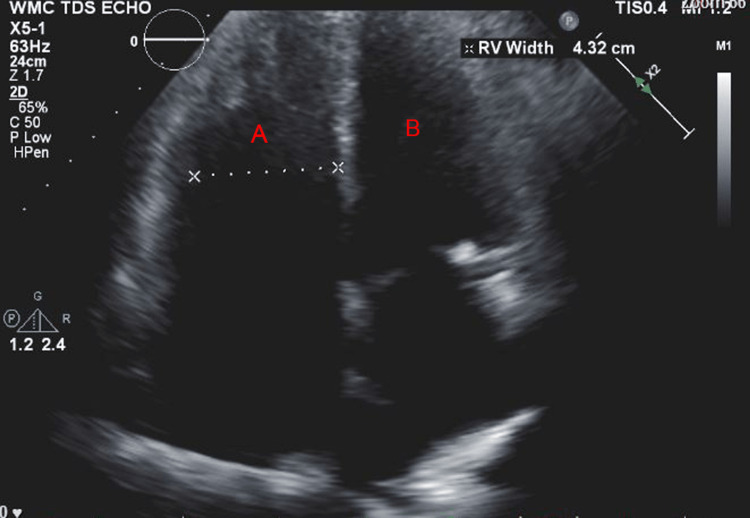
Preoperative TTE apical four-chamber view showing (A) dilated right ventricle with increased wall thickness and (B) left ventricle TTE: transthoracic echocardiogram

**Figure 2:**
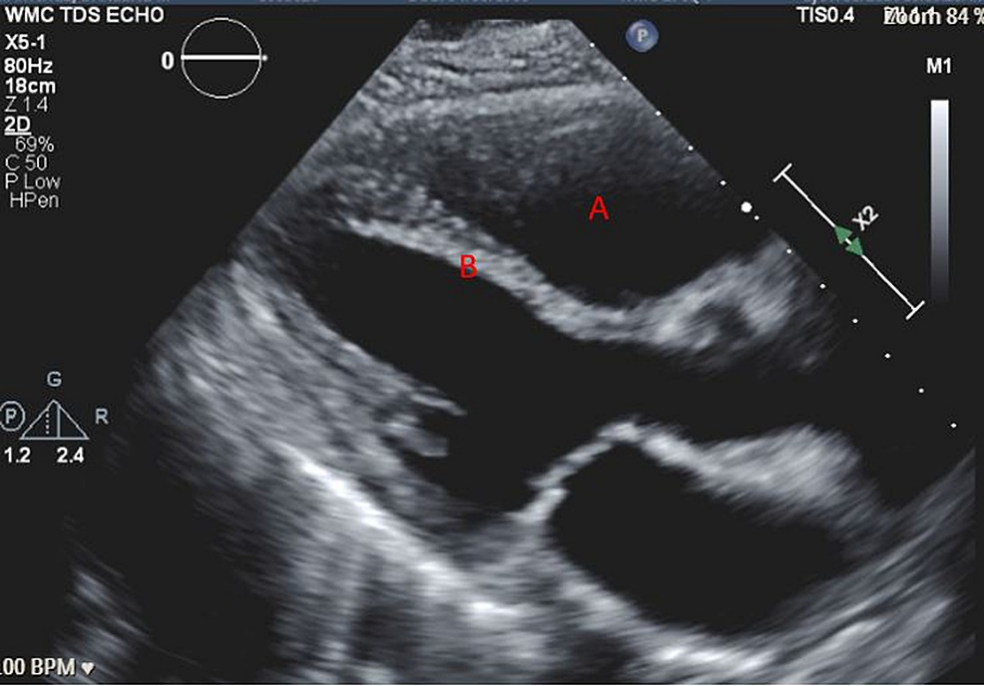
Preoperative TTE parasternal long axis view showing (A) dilated right ventricle and (B) flattened intraventricular septum as evidence of right-sided pressure and volume overload. TTE: transthoracic echocardiogram

**Figure 3:**
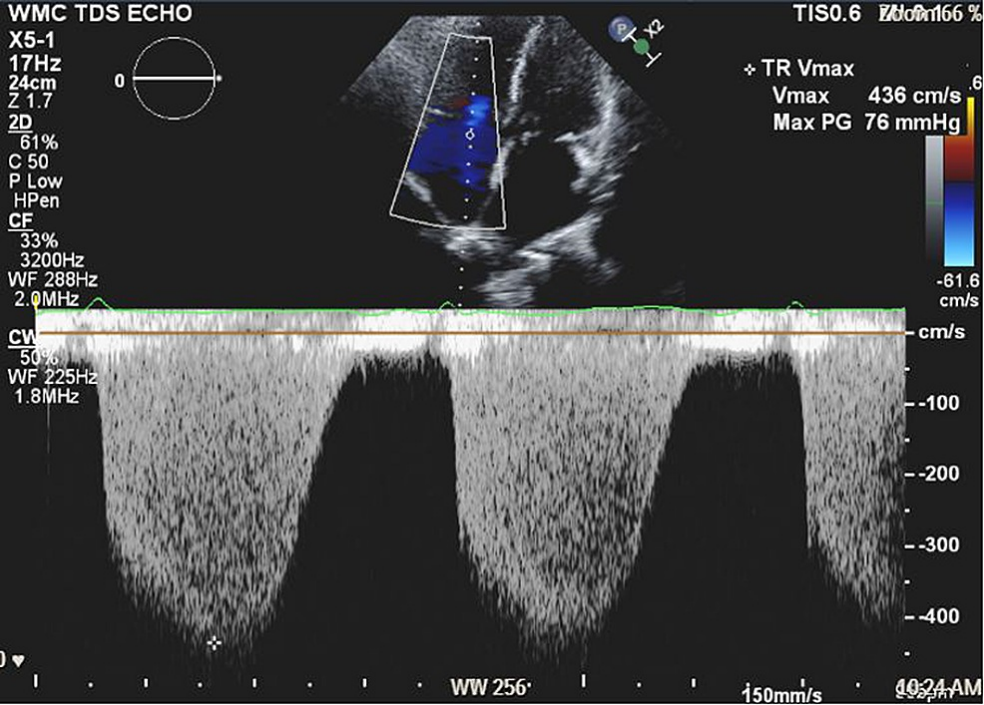
Preoperative TTE apical-chamber view with tricuspid regurgitation and dilated right ventricle. Right ventricular systolic pressure (RVSP) = 76 mmHg + right atrial pressure (RAP). TTE: transthoracic echocardiogram

A 57-year-old woman (American Society of Anesthesiologists (ASA) 4, body mass index 19) who underwent multiple surgeries on her left hip presented with chronic methicillin-resistant *Staphylococcus aureus* (MRSA) infection. She was scheduled for left hip irrigation and debridement, removal of antibiotic cement spacer, and either stage II revision arthroplasty or placement of a repeat antibiotic spacer based on intraoperative findings and frozen sections. Her past medical history was only significant for scleroderma and PH (New York Heart Association (NYHA)/World Health Organization (WHO) Class 1). Her PH was managed by treprostinil 80 ng/kg/min subcutaneously (SC), riociguat 2.5 mg every eight hours, and torsemide 20mg every 12 hours. Preoperative transthoracic echocardiogram (TTE) showed an increased estimated pulmonary arterial systolic pressure (PASP) of 80-90 mmHg, with dilatation of right ventricle (RV) with moderately reduced systolic function of the RV (Figure 1 and Figure 2). There was also flattening of the interventricular septum during both systole and diastole suggestive of RV pressure and volume overload (Figure 2) and tricuspid regurgitation (Figure 3). The left ventricle (LV) had preserved ejection fraction (EF) and grade I diastolic dysfunction as impaired relaxation. Right heart catheterization showed a right atrial (RA) pressure of 4-5 mmHg, RV pressure of 83/7 mmHg, pulmonary arterial (PA) pressures of 81/27 mmHg with a mean of 50 mmHg, cardiac output (CO) 4.51 L/min, and cardiac index (CI) 2.3 L/min. Other perioperative laboratory tests were unremarkable.

She was brought to the operating room (OR) and standard ASA monitors were placed. Oxygen was delivered via a non-rebreather facemask at 10 L/min. A pre-procedural right radial arterial line and two 18-G peripheral venous lines were placed. Baseline blood pressure (BP) was 140-150/80-90 mmHg, heart rate (HR) 80-90 beats per minute (bpm), and oxygen saturation (SpO2) 100%. Vasopressin infusion was started preemptively at 0.04 units/min.

Combined spinal-epidural anesthesia was planned for surgical anesthesia. Prior to CSE placement, 250 ml of crystalloid solution and midazolam 1 mg were given. CSE was placed at the L4-L5 interspace. Bupivacaine 0.75% 1 mL was administered intrathecally, followed by incremental 2% lidocaine (5 mL/bolus, two boluses) via the epidural catheter to optimize an adequate anesthesia level with minimal hemodynamically changes. Two boluses of ketamine 25 mg were given intravenously (IV) and followed by dexmedetomidine 20 mcg IV when the patient was in a supine position. She was hemodynamically stable and saturated well on spontaneous ventilation (respiratory rate 16-18 breaths per min, arterial oxygen saturation 99%). Immediately after right lateral decubitus positioning, the patient became rapidly hypotensive 60/30 mmHg and tachycardic with HR 120 bpm, then became unresponsive. She was immediately placed back in the supine position. Incremental doses of epinephrine 10 µg were immediately given to the patient through IV with minimal response in BP and HR.

**Figure 4:**
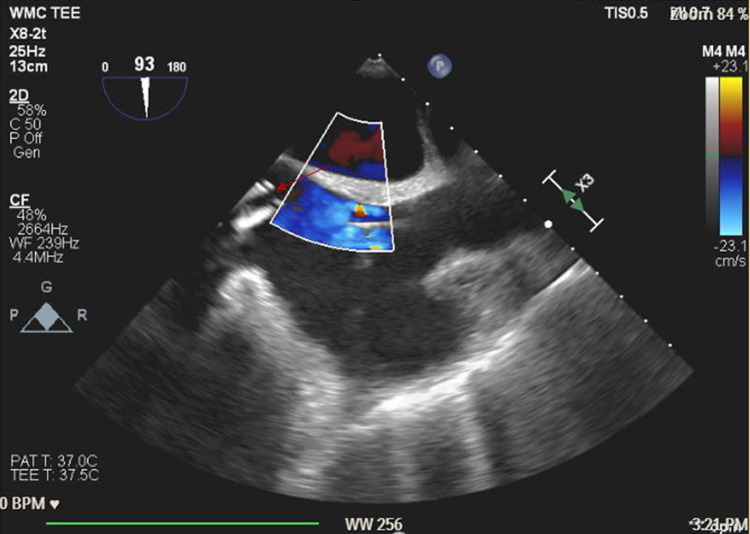
Perioperative TEE (mid-esophageal bicaval view) immediately after VA-ECMO femoral cannulation. Red arrow shows ECMO canula in the atriocaval junction. TEE: transesophageal echo; VA: veno-arterial; ECMO: extracorporeal membrane oxygenation

Advanced cardiovascular life support (ACLS) protocol was initiated. The patient was intubated with a 7.5 endotracheal tube and placed on positive pressure ventilation with 100% O2. Nitric oxide (NO) was started at 40 parts per million (ppm). ACLS was continued and three boluses of epinephrine 1 mg through IV were given with intermittent return of spontaneous circulation (ROSC). Emergent VA-ECMO was placed via left femoral artery and right femoral vein access. A transesophageal echo (TEE) probe was placed immediately and revealed severe RV dilatation, severe RV dysfunction, and no clots in the main pulmonary artery (Figure 4). A Swan-Ganz catheter was placed via the right internal jugular vein. She was transferred to the cardiothoracic intensive care unit (CTICU) for continuous hemodynamic monitoring and support. She was stable with a circuit flow of 4.6 L/min, a pump speed of 4200 revolutions per minute (RPM), mean arterial pressure (MAP) 65-70 mmHg, central venous pressure (CVP) 8-9 cmH2O, and PA pressures 45-57/24-28 mmHg. Treprostinil was switched to 80 ng/kg/min IV and NO was continued at 40 ppm. Vasopressor support was maintained with norepinephrine 0.05 µg/kg/min, epinephrine 0.05 µg/kg/min, and vasopressin 0.04 units/min.

On hospital day two, ECMO was revised to right femoral vein and left axillary artery in preparation for right lateral decubitus positioning and intra-operative left hip manipulation in the OR. Norepinephrine was weaned off, and epinephrine (0.05 µg/kg/min) and vasopressin (0.03 units/min) were continued. On hospital day three, the patient was brought back to the OR, intraoperative TEE revealed slight improvement in RV function. She received left hip irrigation and debridement, antibiotic cement spacer removal, and stage II revision arthroplasty under general anesthesia (GA) with no complications. She was transferred back to CTICU for post-operative care. Serial bedside TTE examinations revealed improvement in RV function. On hospital day five, vasopressin was weaned off and ECMO was successfully removed. Epinephrine 0.02 µg/kg/min was continued for inotropic support of right heart function. On hospital day six, epinephrine was weaned off. She was hemodynamically stable and discharged from the CTICU.

## Discussion

Updated hemodynamic definitions and clinical classification of PH

PH was defined as a mean pulmonary artery pressure (mPAP) >25 mmHg at rest since the first World Symposium on Pulmonary Hypertension (WSPH) in 1973 [9]. Given that the normal mPAP for a healthy individual is 14 ± 3.3 mm Hg, the upper limit (>97.5th percentile) of the normal mPAP is 20 mmHg [10], the increased mortality, and the risk of poor outcome in individuals with borderline mPAP of 21-24 mmHg [11-14], the sixth WSPH in 2018 updated the hemodynamic definition of PH [15]. The task force recommended that PH definition should be changed to mPAP >20 mmHg. However, the level of mPAP is not sufficient to characterize pulmonary vascular disease since it does not differentiate the status of CO or pulmonary artery wedge pressure (PAWP). Therefore, pulmonary vascular resistance (PVR =(mPAP-PAWP)/CO) has been included to define the presence or absence of pre-capillary PH due to elevation of PAWP or CO. The updated hemodynamic definition of PH (mPAP >20 mmHg) will be further subclassified as pre-capillary PH (PAH), isolated post-capillary PH (IpcPH), or combined pre- and post-capillary PH (CpcPH) based on PAWP and PVR (Table 1). The sixth WSPH also updated the clinical classification of PH. Patients who have long-term clinical and hemodynamic response to calcium channel blocks, pulmonary veno-occlusive disease (PVOD), pulmonary capillary hemangiomatosis (PCH), and persistent PH of the newborn syndrome were included as a separate subgroup of group 1 PAH. PH with unclear and/or multifactorial mechanisms was simplified (Table 2).

**Table 1:** Hemodynamic definitions of pulmonary hypertension. mPAP: mean pulmonary arterial pressure; PAWP: pulmonary arterial wedge pressure; PVR: pulmonary vascular resistance; WU: Wood units; PH: pulmonary hypertension Clinical group 1: PAH; Clinical group 2: PH due to left heart disease; Clinical group 3: PH due to lung diseases and/or hypoxia; Clinical group 4: PH due to pulmonary artery obstructions; Clinical group 5: PH with unclear and/or multifactorial mechanisms. Table source: Reprinted from Simonneau et al. [15]. Copyright © 2021 with permission from the European Respiratory Society.

Classification	mPAP	PAWP	PVR	Clinical groups
Isolated pre-capillary PH (PAH)	>20 mm Hg	≤15 mm Hg	≥3 WU	1, 3, 4, and 5
Isolated post-capillary PH (IpcPH)		>15 mm Hg	< 3 WU	2 and 5
Combined pre- and post-capillary PH (CpcPH)			≥3 WU	2 and 5

**Table 2:** Updated clinical classification of pulmonary hypertension. PAH: pulmonary arterial hypertension; PVOD: pulmonary veno-occlusive disease; PCH: pulmonary capillary hemangiomatosis; LVEF: left ventricular ejection fraction; PH: pulmonary hypertension Table source: Reprinted from Simonneau et al. [15]. Copyright © 2021 with permission from the European Respiratory Society.

1 PAH
1.1 Idiopathic PAH
1.2 Heritable PAH
1.3 Drug- and toxin-induced PAH
1.4 PAH associated with:
1.4.1 Connective tissue disease
1.4.2 HIV infection
1.4.3 Portal hypertension
1.4.4 Congenital heart disease
1.4.5 Schistosomiasis
1.5 PAH long-term responders to calcium channel blockers
1.6 PAH with overt features of venous/capillaries (PVOD/PCH) involvement
1.7 Persistent PH of the newborn syndrome
2 PH due to left heart disease
2.1 PH due to heart failure with preserved LVEF
2.2 PH due to heart failure with reduced LVEF
2.3 Valvular heart disease
2.4 Congenital/acquired cardiovascular conditions leading to post-capillary PH
3 PH due to lung diseases and/or hypoxia
3.1 Obstructive lung disease
3.2 Restrictive lung disease
3.3 Other lung disease with mixed restrictive/obstructive pattern
3.4 Hypoxia without lung disease
3.5 Developmental lung disorders
4 PH due to pulmonary artery obstructions
4.1 Chronic thromboembolic PH
4.2 Other pulmonary artery obstructions
5 PH with unclear and/or multifactorial mechanisms
5.1 Hematological disorders
5.2 Systemic and metabolic disorders
5.3 Others
5.4 Complex congenital heart disease

Perioperative outcome and risk factors in patients with PH

Patients with PH are at increased risk for perioperative morbidity and mortality when undergoing cardiac [16-18] and non-cardiac surgery [1-6,19]. Several retrospective [1,2,4,6] and prospective [5] analyses had reported that perioperative mortality ranged from 1-9.7% with morbidity of 24-42% among patients with PH in non-cardiac surgery. Major adverse events were reported [4-6] including right heart failure, delayed tracheal extubation, stroke, myocardial ischemia/infarction, arrhythmia, respiratory failure, sepsis, and renal insufficiency.

A previous study by Ramakrishna et al. had identified independent predictors of short-term morbidity including a history of pulmonary embolism (PE), NYHA functional class ≥II, intermediate- to high-risk surgery, and duration of anesthesia ≥3 h [2]. Right-axis deviation, RV hypertrophy, RV index of myocardial performance ≥ 0.75, right ventricular systolic pressure (RVSP)/systolic blood pressure ≥ 0.66, intraoperative use of vasopressors, and anesthesia when nitrous oxide was not used were associated with early mortality. Recently, Smilomitz et al. reviewed adult patients undergoing non-cardiac surgery from 2004 to 2014 of the Healthcare Cost and Utilization Project’s National (Nationwide) Inpatient Sample (NIS) [3]. The major adverse cardiovascular events (MACCEs) including in-hospital death, myocardial infarction, or ischemic stroke were increased in patients with PH when compared with patients without PH (8.3% vs 2.0%), with the highest surgery-specific risks related to neurosurgery (13.0% vs 4.1), thoracic surgery (12.2% vs 5.6%), vascular surgery (11.2% vs 7.3%) and general surgery (9.3% vs 2.7%) [3]. Memtsoudis et al. identified 3543 PH patients undergoing primary THA and total knee arthroplasty (TKA) from NIS data from 1998 to 2006 and revealed around four-fold increased risk for perioperative mortality after THA (2.4% vs 0.6%) and TKA (0.9% vs 0.2%) as well as increased incidence of acute respiratory distress syndrome (ARDS), PE, and deep vein thrombosis (DVT) in patients with PH [19]. Independent risk factors associated with complications after THA or TKA were the presence of PH, increasing age, male gender, and emergent admission [19].

Pre-operative assessment

Given the significant morbidity and mortality associated with PH, perioperative management should be provided by a multidisciplinary team including anesthesiologists, surgeons, cardiologists, and pulmonologists, and psychological and social worker support [20]. The pre-operative assessment should include evaluating the type of surgery, severity of PH, and comorbidities [21].

High-risk surgery is associated with increased mortality and morbidity in PH patients, especially in emergency surgery, major organ procedures, rapid blood loss, and long operation time [22]. Current international guidelines had recommended risk assessment for patients with PH (Table 3) [23-25]. It is recommended to assess the severity of PH patients with detailed medical history, physical examination, exercise tests, biochemical markers, and echocardiographic and hemodynamic evaluation [24]. Some clinical symptoms are not specific, including shortness of breath, fatigue, chest pain, and syncope. While jugular venous distension, hepatomegaly, ascites, and lower extremities edema may indicate signs of RV dysfunction/failure. Digit clubbing and sclerodactyly may suggest the severity of interstitial lung disease and prolonged hypoxemia. An electrocardiogram (ECG) is sensitive to detect lateral or inferior myocardial ischemia and arrhythmia. Supraventricular arrhythmia, especially atrial flutter or atrial fibrillation has been reported in advanced PH patients, which may result in clinical deterioration and RV failure [26]. TTE is recommended as an important non-invasive tool to visualize the RV size and RV function as well as estimate tricuspid regurgitation velocity (TRV) and right atrial pressure (RAP) with continuous wave Doppler [27]. Recent studies have shown that cardiac magnetic resonance (CMR) imaging is a more accurate non-invasive tool than echocardiography to assess RV morphology, RV function, stroke volume, and CO [28]. Right heart catheterization (RHC), however, remains the gold standard to establish the diagnosis and evaluate the severity of PH with valuable information of PA, PAWP, PVR, and pulmonary vasoreactivity [24,29], and is highly recommended in advanced PH patients before surgery [22].

**Table 3:** Risk assessment and mortality prediction in pulmonary hypertension. RA: right atrial; RV: right ventricular; LV: left ventricular; EF: ejection fraction; WHO: World Health Organization; RVESVi: RV end-systolic volume index; LVEDVi: left ventricular end-diastolic volume index; SvO2: mixed venous oxygen saturations; CPET: cardiopulmonary exercise test; CI: cardiac index; RAP: right atrial pressure; 6MWD: six-minute walk distance; VO2: oxygen consumption; VE/VCO2: ventilatory equivalents for carbon dioxide BNP: brain natriuretic peptide; NT-proBNP: N-terminal BNP Table Source: Reprinted from Price et al. [25]. Copyright © 2021 with permission from Elsevier Ltd.

	Low Risk	Intermediate Risk	High Risk
Clinical Assessment			
Right heart failure	None	None	Present
Progression of symptoms	None	Slow	Rapid
Syncope	None	Occasional	Recurrent
Chest pain	None	Occasional	Recurrent
Arrhythmia	None	Occasional	Recurrent
WHO functional class	I/II	III	IV
Imaging and Hemodynamics			
Echocardiography	Preserved RV function. RA area < 18 cm^2^. No pericardial effusion	Impaired RV function. RA area 18-26 cm^2^. No or minimal pericardial effusion	Impaired RV function. RA area >26 cm^2^. Pericardial effusion present
Cardiac magnetic resonance	High RVEF >54%, normal RVESVi, normal LVEDVi	Reduced RVEF (37-54%), increased RVESVi, decreased LVEDVi	Reduced RVEF (<37%), increased RVESVi, decreased LVEDVi
Right heart catheterization	RAP <8 mmHg, CI> 2.5 L/min/m^2^, SvO2 >65%	RAP 8-14 mmHg, CI 2-2.4 L/min/m^2^, SvO2 >60-65%	RAP >14 mmHg, CI <2 L/min/m^2^, SvO2 <60%
Exercise Capacity			
6MWD	> 440 m	165-440 m	< 165 m
CPET	Peak VO_2_ >15 ml/min/kg (>65% predicted), VE/VCO_2_ slope <36	Peak VO_2_ 11-15 ml/min/kg (35-65% predicted), VE/VCO_2_ slope 36-44.9	Peak VO_2_ <11 ml/min/kg (<35 predicted), VE/VCO_2_ slope >45
Biomarkers			
BNP	< 50 ng/L	50-300 ng/L	>300 ng/L
NT-pro BNP	< 300 ng/L	300-1400 ng/L	>1400 ng/L

Given that decreased diffusing capacity of the lung for carbon monoxide (DLCO) in patients with PH is associated with higher mortality [30,31], pulmonary function tests and arterial blood gas analysis should be considered especially for IPAH and group 3 PH patients. Ventilation/perfusion (V/Q) and CT pulmonary angiography remain the major diagnostic tools for chronic thromboembolic PH (CTEPH) [32]. CMR also can be used to determine segmental occlusions of the pulmonary arteries in a CTEPH patient who is contraindicated for CT angiography (CTA) [33].

A modified NYHA/WHO functional classification system remains one of the most powerful predictors of survival and follow-up [34,35]. Either the six-minute walking test (6MWT) [36,37] or cardiopulmonary exercise testing (CPET) [38,39] provides information on cardiovascular and respiratory response exercise capacity and obtains a reliable evaluation of functional status and RV function. However, 6MWT or CPET may not be performed pre-operatively due to comorbidities that limit patients’ ability to ambulate.

A basic laboratory workup should include complete blood counts and a comprehensive metabolic panel. Prothrombin time and international normalized ratio should be performed while the patient is on oral anticoagulants [24,40,41]. N-terminal pro-brain natriuretic peptide (NT-proBNP) levels correlate with myocardial dysfunctions, reflect pulmonary hemodynamics, and are reliable predictors of prognosis in PH patients [42,43].

Choice of anesthesia for non-cardiac surgery

GA and regional anesthesia (RA) have been used in patients with PH undergoing non-cardiac surgery. The general principle of anesthesia management is to maintain RV function/CO with adequate preload, contractility, and systemic vascular resistance (SVR) and avoid increases in PVR from hypoxia, hypercarbia, acidosis, agitation, pain, and hypothermia. However, there are no controlled studies or practice guidelines advising the use of GA or RA for PH patients [44,45]. The decision in terms of anesthesia technique depends on the type of surgery, RV function/PA pressure, and comorbidities.

Induction of GA is one of the most precarious moments in the care of these patients as a sudden decrease in CO with an increase in PVR and SVR in response to sympathetic activation during tracheal intubation, which may result in a pulmonary hypertensive crisis, RV failure, and subsequent circulatory collapse. Therefore, a balanced technique with increased opioid dosing is often employed [25].

Perioperatively, it was recommended that invasive monitoring includes pre-induction/pre-procedural arterial line in patients with severe PH [21]. In patients with preserved LV function, CVP monitoring may reflect RV preload, RV filling pressures, and global RV performance. Although a central line for CVP monitoring may have limited applications, a pulmonary arterial catheter may be more useful for monitoring intraoperative pulmonary pressures and providing continuous CO monitoring [21]. Intraoperative TEE may further be indicated in patients with severe PH. It allows beat-to-beat monitoring of LV and RV function and ventricular filling, guides intraoperative fluid therapy, and detects ventricular ischemia by identifying regional wall motion abnormalities [46]. Close monitoring of the tricuspid valve with or without dynamic tricuspid regurgitation as well as estimations of systolic, mean, and diastolic pulmonary pressures may also be possible. Finally, both quantitative and qualitative evaluations of RV function can be performed. However, this requires GA along with an experienced TEE operator [21].

GA with endotracheal intubation allows anesthesiologists to maintain a patent airway, maximize oxygenation by mechanical ventilation, precise control of the composition of inhaled oxygen, volatile anesthetics, inhaled pulmonary vasodilating agents, and perform TEE intraoperatively. Low-tidal-volume positive pressure ventilation (PPV) (5-7 ml/kg) with minimal positive end-expiratory pressure (PEEP) has been recommended to avoid overinflation of alveoli. Goals for mechanical ventilation and tracheal intubation are again targeted at maintaining an adequate level of sedation and sympathetic activation as well as preventing hypercapnia, respiratory acidosis, hypoxia, and increased PVR [47,48]. However, physiological changes during GA may increase PVR. PPV with or without PEEP increases intrathoracic pressure, which may result in decreases in venous return and RV preload [49,50]. GA-induced atelectasis may lead to perioperative hypoxia and in turn cause increases in PVR [51,52]. PH caused by interstitial lung disease has a high incidence of developing barotrauma from mechanical ventilation [53]. Volatile and intravenous anesthetics such as isoflurane [54], sevoflurane [55], and propofol [56] have minimal effect on sweating thresholds but greatly reduce the vasoconstriction and shivering thresholds, which impair the thermoregulatory control and raise the concerns of perioperative hypothermia [57]. In vivo hemodynamic monitoring has shown that mild hypothermia resulted in increases in SVR and PVR [58], which may aggravate the RV dysfunction.

Vasodilator therapy has been used intraoperatively to reduce the PA pressure and decrease pulmonary shunt [48]. Inhaled selective pulmonary vasodilators are preferred over intravenous routes due to their minimal effect on the systemic circulation [47,59,60]. Vasopressors are usually required while a patient is under GA. Most commonly norepinephrine, vasopressin, and phenylephrine are used. Although vasopressors have the potential to increase PVR, their use by an experienced anesthesiologist is necessary to maintain coronary perfusion in the setting of decreased SVR from the anesthetics. Historically, vasopressin is preferred as it can increase SVR without minimal effects on PVR. Inotropes such as dobutamine and dopamine may also be used to improve RV performance. However, they may predispose patients to develop tachycardia and/or other arrhythmias, which may limit their application [25].

Neuraxial anesthesia, deep plexus, or peripheral nerve blockade with or without sedation have been provided as surgical anesthesia for patients with PH in non-cardiac surgery. Maintenance of the native airway during RA with adequate oxygenation with a face mask or non-rebreather mask will avoid GA-induced atelectasis [52] and mechanical ventilation-induced intrathoracic pressure elevation [49,50,61]. Neuraxial blockade either spinal or epidural anesthesia provides reliable surgical anesthesia. However, due to rapid onset and sympathetic blockade, single shot or fixed doses of spinal anesthesia is not recommended since it can produce dramatic reduction of cardiac preload with significant decrease in CO and reflex tachycardia, which leads to myocardial ischemia and RV failure [62]. In patients with severe hypovolemia, spinal anesthesia may induce severe bradycardia, which may rapidly progress to asystole due to the Bezold-Jarisch reflex [63]. Therefore, catheter-based neuraxial techniques such as continuous spinal anesthesia, continuous epidural anesthesia, and CSE with incremental dose titration are potential alternatives to provide effective surgical anesthesia and post-op analgesia with minimal hemodynamic changes. An earlier cohort study of 65 parturients with congenital heart disease or PH by Maxwell et al. revealed that epidural or CSE were predominantly used for cesarean section due to favorable maternal and neonatal outcomes [64]. A recent retrospective cohort study by Tsukunaga et al. reviewed 263 cesarean sections with cardiovascular disease including PH from 1994 to 2019, catheter-based neuraxial anesthesia was performed in 214 (81.3%) parturients with less hemodynamic instability, postpartum heart failure or need for neonatal intubation [65]. Continuous spinal anesthesia has been used successfully in hip arthroplasty [66-71] and cesarean section [72,73] in patients with severe PH or aortic stenosis and demonstrated minimal hemodynamic changes and adequate post-op analgesia. Besides neuraxial techniques, single shot or continuous lumbar plexus and sciatic nerves blockade have been used for lower extremities surgeries [74]. At our institution, lumbar plexus and single shot transgluteal sciatic blocks with light sedation had provided adequate surgical anesthesia in elderly patients with severe PH or severe aortic stenosis for THA.

However, when patients are not candidates for neuraxial or deep plexus blockade due to the time and dose of systemic anticoagulation or other medical issues, peripheral nerve block has been approved as a great alternative for orthopedic, vascular, and general surgery. Seyfarth et al. reviewed 16 patients with PH undergoing major orthopedic surgery for perioperative morbidity and mortality from 2011 to 2014; combined moderate GA and femoral nerve block were used for THA, and combined GA and sciatic nerve block were used for TKA [71]. Combination of GA and peripheral nerve block minimized intraoperative hemodynamic instability and vasodilation from anesthetics and ensured post-operatively adequate pain control. Recently truncal nerve blocks have been used in patient with PH undergoing abdominal surgery. Duggan et al. had reported a patient with severe PH, right heart failure and bilateral lower extremities paralysis due to Pott’s disease who presented for fulguration of anal condyloma and diverting colostomy and was not a candidate for neuraxial anesthesia due to sacral debubitus ulcer [75]. The patient was able to tolerate subxiphoid incision, electrocautery, and diverting colostomy under bilateral rectus sheath blockade, sedation with dexmedetomidine infusion, and minimal intraoperative opioids. It was reported by Boretsky et al. that bilateral quadratus lumborum and rectus sheath blockade had been used as low-risk analgesia to prevent pulmonary hypertensive crisis in a five-month-old infant with severe PH who underwent laparoscopic cholecystectomy [76]. The patient had shown well hemodynamic stability, oxygen saturation, and no changes to sedation during the post-operative 24-hour period.

Combination of sedation and RA has provided advantages when managing high-risk patients undergoing non-cardiac surgery, but still requires constant vigilance and dose titration for sedation to avoid attenuation of respiratory drive and hypoxia-induced pulmonary hypertensive crisis [22,77]. In the event of inadequate surgical blockade or failed RA, there are no controlled studies to show how to choose appropriate options to rescue failed block in patients with PH. When options are limited and salvage block is not feasible, emergency converting to GA might be necessary [78,79]. Recently studies also have shown that high-thoracic epidural anesthesia induced sympathectomy reduces RV contractile performance and limits the capacity of RV to adapt to the increases of RV afterload, which may lead to decreases in CO and cardiovascular collapse in patient with PH [80-82].

Right ventricular failure and ECMO

The right ventricle is triangularly shaped and is often described as a crescent in cross section. The thin free wall undergoes a longitudinal and sequential contraction. Compared to the LV, it has a lower EF, approximately 40-45%, with approximately 40% of the contractility derived from the septum [83,84]. Therefore, in order to generate the same stroke volume and CO, it maintains a higher end diastolic volume. The RV is exquisitely afterload sensitive; however, it can better tolerate volume overload. As a result, RV stroke volume decreases in a linear fashion with an acute increase in pulmonary vascular resistance. As there is progressive RV dilatation, the intraventricular septum begins to impede in the LV, compromising LV function [83]. Interestingly, the RV can recover even after ischemic injury [84].

Pulmonary hypertensive crisis, as described in our patient, is the most dreaded intraoperative complication due to rapid hemodynamic deterioration. Goals include early recognition of increase in PVR and RV filling pressures with a decrease in CO, and hypotension prior to impending cardiovascular collapse. Therefore, hypotension should be aggressively treated with vasopressors to maintain right coronary perfusion and RV function. Reduction in PVR may be achieved with inhaled nitric oxide, prostacyclin, iloprost, milrinone, and or sildenafil [25]. Inotropes are used to support low CO and systemic hypotension. Dobutamine, dopamine, epinephrine, and milrinone are normally used in the ICU or perioperative period to improve the contractility of RV and LV [85]. However, there is limited evidence to guide inotrope selection in RV failure for PH. Milrinone or dobutamine are often combined with vasopressor to support RV contractility and increase coronary perfusion pressure. Epinephrine is typically used for severe refractory cardiogenic shock; however, it is associated with tachycardia, increased myocardial oxygen consumption and PVR. Novel inotropes such as mitotropes or myotropes use alternative intracellular pathways that are still under investigation and may minimize the adverse effects such as arrhythmia or ischemia [86].

In patients with PH who deteriorate into cardiopulmonary arrest, resuscitation is often difficult and have had limited success [25,84,87]. The use of VA-ECMO as a rescue in cardiopulmonary arrest has been increasing in the United States, as there has been increased availability of membranes and portable circuits, the ability of ECMO to provide left, right, and biventricular support, its ease of implantation intraoperatively, in the catheterization laboratory, or even bedside [7,8,88]. VA-ECMO insertion during cardiopulmonary arrest has revamped its role in initial cardiopulmonary resuscitation (CPR) management [89,90]. Its availability for rapid deployment even in patients who fail to achieve a sustained return of spontaneous circulation also makes it an invaluable therapeutic option. This idea of extracorporeal cardiopulmonary resuscitation (ECPR) should include a no-flow time of less than five minutes with ECPR preparation within 10 minutes, and cannulation within 20 minutes [91].

In our patient, VA-ECMO as part of ECPR was indicated due to cardiogenic shock as the result of RV failure. Other indications involving specific the right ventricle include acute RV failure due to pulmonary embolism, progression of congenital heart disease, acute myocardial infarction, acute myocarditis, and septic cardiomyopathy [88]. It is important to note absolute contraindications including severe, irreversible, noncardiac organ failure, anoxic brain injury, metastatic cancer, irreversible cardiac failure if transplantation or long-term ventricular assist device (VAD) are not considered, and aortic dissection [88]. While the use of VA-ECMO can be used as rescue therapy during a pulmonary hypertensive crisis and/or cardiopulmonary resuscitation, we should be mindful of the final goals of therapy. ECMO does not support RV directly, it has been used as a temporizing measure to provide circulatory support in an ICU setting while the myocardium has time to recover or as a bridge to a decision therapy to achieve stability for end-organ perfusion until more definitive mechanical support therapy or cardiac replacement therapy can be instituted [7,8,88]. However, these patients who are placed on ECMO and do not proceed to transplantation often remain at a high mortality risk [25,83]. There were studies showing that the use of additional mechanical RV support devices such as intra-aortic balloon counterpulsation (IABP) or Impella in VA-ECMO patients was associated with decreased mortality [92,93].

Although VA-ECMO can provide effective mechanical circulatory support and allows for decompression of the RV, it is not specifically tailored towards isolated right heart failure. Specifically, systemic arterial inflow bypasses normal functioning LV or LVAD, resulting increased afterload [83]. Therefore, VA-ECMO is limited in patients with aortic valve insufficiency, which may result in LV distention. And so, strategies can be employed to reduce pulmonary congestion and promote forward flow. These include the addition of inotropic support or the insertion of Impella devices or intra-aortic balloon pumps. Mechanical LV decompression may be achieved via an atrial septostomy to promote LV shunting of blood to the right heart [8,83,88]. Diuretic agents, ultrafiltration, and hemodialysis may also be used to remove fluid from the intravascular space [88]. Complications of VA-ECMO placement include limb ischemia from peripheral cannulation or compartment syndrome, stroke (both ischemic and hemorrhagic), bleeding, infection, and Harlequin syndrome [88]. Anterograde distal limb perfusion and Doppler monitoring may reduce the risk of limb ischemia [83].

## Conclusions

Patients with moderate to severe PH who are scheduled for non-cardiac surgery require complex and well-thought decision making by anesthesiologists, and perioperative planning among a multidisciplinary team to ensure a good outcome. Although there is no clear-cut data on which modality is better, GA, neuraxial anesthesia with catheter, and peripheral nerve blocks were successfully implemented. The anesthesiologist should approach these cases with an understanding of the PH pathophysiology, and possible intraoperative complications based on the modality he or she chose, such as pulmonary hypertensive crisis, which in this case required VA-ECMO rescue by sudden increases in PVR and PA pressure can be triggered by a variety of intraoperative events, such as changes in patient position, hypercapnia, hypotension, and hypoxia. A strategy of how to address these triggers should be formulated prior to the start. While ECMO availability is variable among different institutions, it is important to note that its application for cardiopulmonary arrest, in this case, secondary to right heart failure, remains a temporizing measure until there is recovery of the myocardium or another long-term plan can be instituted.
